# Extrusion and nixtamalization conditions influence the magnitude of change in the nutrients and bioactive components of cereals and legumes

**DOI:** 10.1002/fsn3.1473

**Published:** 2020-02-25

**Authors:** Elijah Heka Kamau, Smith G. Nkhata, Emmanuel Owino Ayua

**Affiliations:** ^1^ Department of Food Science and Nutrition University of Eldoret Eldoret Kenya; ^2^ Department of Food Science and Nutrition Jomo Kenyatta University of Agriculture & Technology Nairobi Kenya; ^3^ Department of Agro‐food Processing Natural Resources College Lilongwe University of Agriculture and Natural Resources Lilongwe Malawi

**Keywords:** antinutritional factor, extrusion, nixtamalization, nutrients, phytochemicals

## Abstract

Cereal and legume diets make up the bulk of caloric sources for a majority of households in the developing world. They contain macro‐ and micronutrients as well as phytochemicals embedded as one matrix. Some phytochemicals are antinutritional factors which can bind nutrients thereby hindering their bioavailability. While there are other methods that can be used to enhance nutrient utilization from such foods, we summarize how food processing methods such as extrusion and nixtamalization are employed to break the food matrix and release these nutrients. Both extrusion and nixtamalization can break down complex carbohydrates into simpler, more soluble forms while at the same time inactivating or denaturing protein inhibitors and other antinutritional factors. Such disruptions of complexes within the food matrix are essential for harnessing optimum nutritional and health benefit from these foods. We present mechanistic approaches explaining how these processes enhance nutrient and mineral bioavailability and phytochemical bioactivity while minimizing the undesirable effects of antinutritional factors that coexist in the complex food matrix.

## Graphical abstract

Extrusion and nixtamalization are employed to break the food matrix and release nutrients. Both processes break down complex carbohydrates into simpler, more soluble forms while at the same time inactivating or destroying protein inhibitors and other antinutritional factors. Disruptions of complexes within the food matrix are essential for harnessing optimum nutritional and health benefit from these foods.

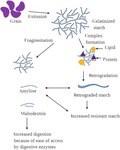

## 1 INTRODUCTION

Through the evolution of the human diet, diversification of the food sources meant that man had to adopt strategies aimed at making food more palatable, attractive with improved organoleptic properties (Fabri & Cosby, 2016; Hotz & Gibson, 2007). These approaches are particularly relevant in times of seasonal variations and in a changing climate which brings about lean seasons and those of peak production. Consequently, adoption of food processing and preservation methods seems handy in enhancing nutrient availability and also extending shelf life of product. While traditional food processing methods such as fermentation and germination have been reported to increase bioaccessibility and ultimate bioavailability of nutrients (El‐Hag, El‐Tinay, & Yousif, 2002; Hubert, Berger, Nepveu, Paul, & Dayde, 2008; Nkhata, Ayua, Kamau, & Shingiro, 2018), there are other processing techniques serving similar roles such as extrusion and nixtamalization.

**Figure 1:**
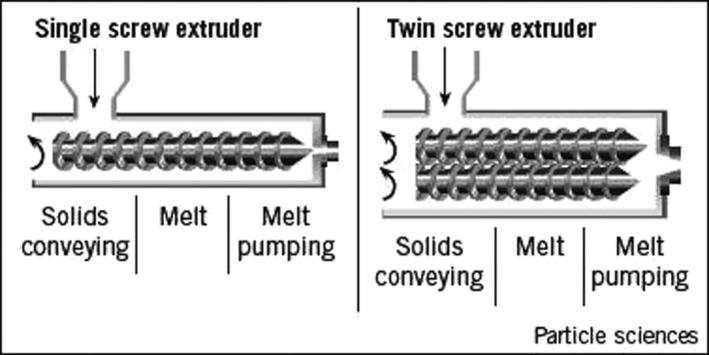
A cross section of single screw (left) and twin screw (right) extruder barrels. Adopted from PTFE machinery (PTFE, 2017)

Extrusion is a heat‐cooking process that is increasingly gaining application in food processing largely due to its efficiency (Sharma, Gujral, & Singh, 2012; Ti et al., 2015) besides its continuous system associated with high productivity (Singh, Gamlath, & Wakeling, 2007). The extruder consists of hopper to feed the extruder with raw materials and ingredients, a screw inside the barrel to create shearing forces on the food mixture, and a die, which is a small opening through which the mixture is pushed out to produce a particular shape of the final product (Figure 1). An extruder can be a either single screw or multiple screw system of which the twin screw is most commonly used (PTFE, 2017).

Since the mid‐1930s, extrusion has continually evolved into one of the crucial food processing technologies applied in production of breakfast foods, ready‐to‐eat snacks, and other textured foods (Brennan, Brennan, Derbyshire, & Tiwari, 2011; PTFE, 2017; Stojceska, Ainsworth, Plunkett, & Ibanoglu, 2009). During extrusion, food matrix is subjected to high temperature (100–150°C) for a short time (Awolu, Oluwaferanmi, Fafowora, & Oseyem, 2015; Cheftel, 1986) during which the food is exposed to shear forces and pressure resulting in alteration of the chemical characteristics, microstructure, and macroscopic shape with intense mixing to attain homogenization and dispersion of ingredients (Anton, Fulcher, & Arntfield, 2009). Consequently, the nutritional value and composition of the food are affected (Bjorck & Asp, 1983; Cheftel, 1986; Singh et al., 2007).

On the other hand, nixtamalization is a maize processing method which involves boiling the corn in water containing lime (calcium hydroxide) at a concentration range of 1%–5% (Gomez, Waniska, & Rooney, 1991). The boiled grain is then steeped overnight before it is washed and ground into corn *masa* (Dombrink‐Kurtzman, Dvorak, Barron, & Rooney, 2002; Gomez et al., 1991). The steeped maize or grain is referred to as nixtamal, while the cooked steep liquid is the *nejayote* (Sefa‐Dede, Cornelius, Sakyi‐Dawson, & Afoakwa, 2004). Nixtamalization is an ancient method practiced by the Aztecs (Sefa‐Dede et al., 2004) and is popularly used in Mexico, Guatemala, and other Latin American countries where it accounts for about 70% of the total calories in the diet through preparation of staples such as tortillas, tortilla chips, taco shells, corn chips, tamales among others (Gomez et al., 1991; Mendez‐Montealvo, García‐Suárez, Paredes‐López, & Bello‐Pérez, 2008; Rojas‐Molina et al., 2007). This process is gradually gaining traction in the USA and other Mesoamerican countries (Fernandez‐Munoz et al., 2007).

## 2 EXTRUSION

### 2.1 Effect of extrusion on minerals and antinutritional factor complexes

Owing to the stability of minerals during extrusion (Camire, Camire, & Krumhar, 1990), relatively few studies have been carried out on the effects of extrusion on minerals (Singh et al., 2007). The few studies carried out largely indicated that extrusion does not have much effect on composition of minerals in extrudates. For instance, in a study to determine the structure and functional properties of pea and kidney beans, Alonso, Rubio, Muzquiz, and Marzo (2001) found no significant change in mineral composition with the exception of iron content which was enhanced. This was attributable to the wearing of the extruder screws during extrusion process. Nevertheless, extrusion led to a significant increase in the apparent absorption of the iron, copper, and phosphorus (Alonso et al., 2001). Extrusion of broken rice with incorporation of wheat bran at 300 r.p.m screw speed, 93–97°C outlet temperature, and 27 kg/hr feed rate in a 5/32 inch die size led to an increase in phosphorus, iron, calcium, and copper (Singh, Chauhan, Suresh, and Tyagi (2000). It has been suggested that the increase in minerals during extrusion can be because of the water used to equilibrate the food to be extruded as well as those from the extruder barrel (Singh et al., 2000, 2007).

Extrusion can enhance the absorption of minerals through deactivation of absorption inhibitors by high temperatures (Alonso et al., 2001). This is because extrusion leads to hydrolysis of phytates, and this phenomenon was put forth as the reason for the increased mineral availability in peas and kidney beans extruded at high temperatures (Alonso et al., 2001). At low temperatures, extrusion reduced phytate content in wheat flour (Singh et al., 2007), but had no effect in legumes (Lombardi‐Boccia, Lullo, & Carnovale, 1991). Reorganization of dietary fiber which can be greater at higher temperature but low moisture extrusion combination to increase the soluble fiber (Bjorck & Asp, 1983; Cheftel, 1986) could also explain the increased mineral absorption as a result of opening up the fiber matrices and increasing fermentation in the colon.

Extrusion at high temperature was reported to reduce antinutritional factors including lectins, trypsin inhibitors, oxalates, and tannins (Kuar, Sharma, Singh, & Dar, 2015; Nikmaram et al., 2017). There was a 37%, 54%, and 72% reduction in oxalates, phytic, acid and trypsin inhibitors, respectively, when cereal bran was extruded at temperature between 115 and 165°C (Gualberto, Bergman, Kazemzadeh, & Weber, 1997). Similarly, phytic acid and the activity of trypsin inhibitors were reduced in extruded peas and kidney beans (Hejdysz, Kaczmarek, & Rutkowski, 2016; Singh et al., 2007). Extrusion at temperatures in the range of 140–180°C, extruder screw speeds of 150–120 rpm, and feed moisture of 14%–22% was effective in reducing tannin, phytic acid, and trypsin inhibitors in lentil splits by 98, 99, and 99%, respectively (Rathod & Annapure, 2016). Therefore, extrusion technology could be used to increase the bioaccessibility of minerals through destruction of mineral–antinutritional factor complexes (Lopez, Gordon, & Fields, 1983), which can help in addressing micronutrient deficiencies. The challenge remains the high cost of extrusion as most developing countries where micronutrient deficiencies remain high may not afford the purchasing and running cost of extruders.

### 2.2 Effect of extrusion on protein digestibility and bioavailability

Heat and mechanical forces can easily denature proteins, which are either detrimental or desirable depending on their intended use. Protein digestibility is the main indicator of protein quality of foods (FAO/WHO/UNU, 1985 1985). Heat and shear force to which foods are subjected during extrusion improve the availability of proteins in different ways (Cheftel, 1986). One way is the inactivation of antinutritional factors such as phytates, tannins, hemagglutinins, and trypsin inhibitors which leads to increased protein bioavailability (Fabbri & Cosby, 2016; Fapojuwo, Maga, & Jansen, 1987; Singh et al., 2007; Ti et al., 2015). Most of the antinutritional factors such as lectins and hemagglutinins which can either inhibit the enzymes involved in proteins digestion or complex proteins are destroyed as the foods are extruded (Nikmaram et al., 2017). For example, greater destruction of trypsin inhibitors can be attained by extrusion at elevated temperatures or by increasing residence time when extrusion is done at lower temperatures (Ti et al., 2015). For instance, in extrusion of wheat flour, increase in extrusion temperatures led to higher inactivation of protease inhibitors with consequent increase in protein digestibility (Singh et al., 2007). Increase of extrusion temperatures to 143°C led to destruction of 57% of trypsin inhibitor but the product had the maximum protein efficiency ratio (PER) which is used to determine the protein quality of a food (Bjorck & Asp, 1983). Hemagglutinins are inactive at temperatures around 80–90°C. However, when heated at 100°C for 5 min their activity was eliminated (Alonso et al., 2001) suggesting that achieving optimal extrusion temperatures is critical to getting desired effects. Therefore, determining optimal extrusion conditions for various parameters (antinutritional factors) that will result in cereal and legume products with higher nutritional value is necessary.

Besides temperature, the shear forces applied during extrusion can open the proteins structure and enhance digestibility (Bhatnagar & Hanna, 1985). It was reported that thermal unfolding of proteins and inactivation of trypsin inhibitors led to improved digestibility and bioavailability of sulfur amino acids (Cheftel, 1986) due to increased surface area upon which enzymes act (Colonna, Tayeb, Mercier, Mercier, Linko, & Harper, 1989). Nevertheless, extrusion can denature protein and promote Maillard reaction (Reddy, Pierson, Sathe, & Salunkhe, 1985) that can lead to loss of essential amino acids including lysine, arginine, histidine, cysteine, methionine, and tryptophan (Areas, Rocha‐Olivieri, & Marques, 2016).

### 2.3 Effect of extrusion on carbohydrates

**Figure 2:**
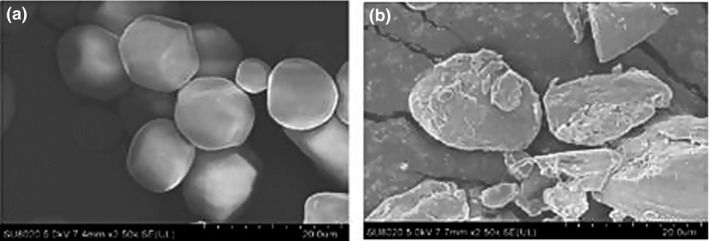
Changes in starch upon extrusion of corn starch: A represents raw corn starch while B is the extruded corn starch as observed in scanning electron micrograph at magnifications of 2500× (Adopted from: Yan et al., 2019)

The effect of extrusion is dependent on the type of grains or legumes. Extrusion increased glucose, galactose, and stachyose in extruded chickpea and glucose, maltose, and stachyose in lentils (Berrios, Morales, Camara, & Sanchez‐Mata, 2010). Starch, the predominant carbohydrate in cereals and grains loses its crystalline structure, swells, and becomes irregular upon extrusion (Cheftel, 1986; Yan et al., 2019) (Figure 2). This is accompanied by partial or complete release of amylose which may alter viscosity. These changes are dependent on the combination of extrusion factors such as moisture, temperature, residence time, and shear force. While more studies need to be done on the precise parameter combinations needed to achieve these modifications, work by Liuko, Colonna, and Mercier (1981), shows that total gelatinization of starch is attained at 120°C and moisture content of 20%–30%. Nevertheless, if higher temperatures are attained, gelatinization can even be achieved at 10%–20% of moisture (Cheftel, 1986).

Yan et al. (2019) also observed an increase in swelling power in extruded compared to non‐extruded corn starch likely due to amylose leaching during extrusion. Extrusion of starches has been reported to favor decrease of amylopectin peaks with subsequent increase in amylose peaks as observed using size exclusion chromatography (Vanier et al., 2016). These authors observed greater amylopectin fragmentation on starches with low initial amount of amylose upon exposure to extrusion shear. This concept has been attributed to be one of the reasons for the increase in starch digestibility in extruded pure starches as it opens starch granules and enhances gelatinization consequently leading to the starches being susceptible hydrolyzing enzymes (Yan et al., 2019). During extrusion, there is partial hydrolysis of amylose and amylopectin into maltodextrins occasioned by the high shear forces (Singh et al., 2007). Starch hydrolysis is faster in cereals and legume flours compared to purified starches. This could be as a result of the presence of amylases which could be active during the initial stages of extrusion (Cheftel, 1986). Nevertheless, the limit for dextrose equivalent after extrusion stands at 3 but this could be enhanced if the flours contained indigenous di‐ or oligosaccharides or if the pH is lowered during extrusion (Cheftel, 1986).

**Figure 3:**
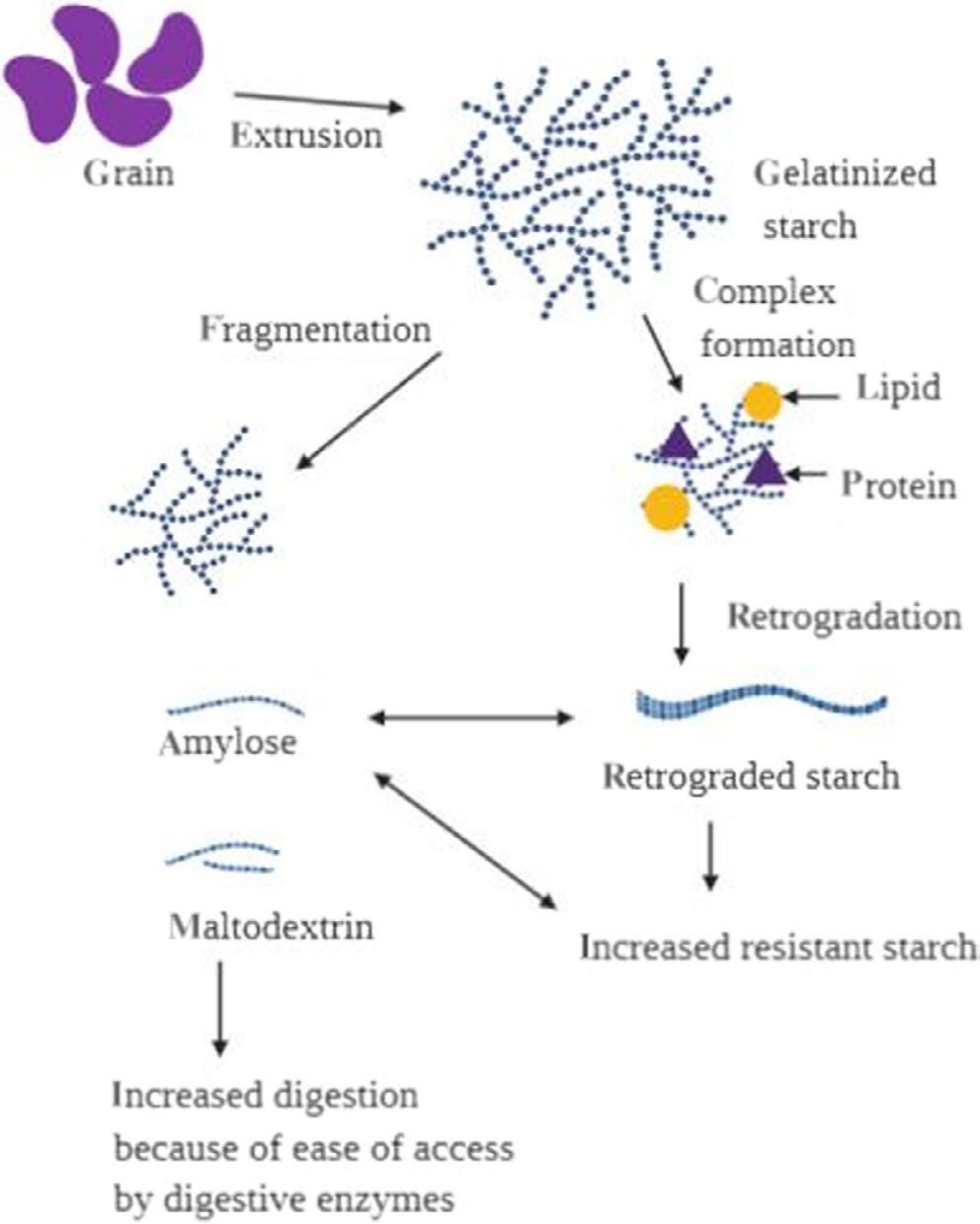
Plausible mechanism by which extrusion cooking increases digestion by opening the polymer molecules enabling access by digestive enzyme. The figure also demonstrates how extrusion could cause carbohydrate–lipid–protein complexes leading to creation of resistant starch. Illustration created by BioRender program

Increase in starch digestibility with extrusion could be due to partial gelatinization, starch polymer fragmentation, and depolymerization of starch allowing for more digestive enzymes access (Figure 3). It was reported that extrusion process can increase digestible carbohydrate in cereals up to threefold compared to their raw and unextruded counterparts (Brennan, Merts, Monro, Woolnough, & Brennan, 2008). The increase in starch digestibility is undesirable because it can result in food with higher glycemic responses (Vinoy et al., 2013) than unprocessed foods. However, as depicted in Figure 3, it is worth mentioning that starch–lipid complexes can also occur during extrusion (Bhatnagar & Hanna, 1994) leading to creation of resistant starches which can lower starch digestibility leading to production of foods which can stimulate laxation, lower glycemia, promote the production of short chain fatty acids, increase solubility and absorption of minerals, and may offer protective properties against diabetes and colorectal cancer (Raignold, Ezekiel, & Raigond, 2015).

Due to starch gelatinization and partial hydrolysis, extrusion is useful in preparation of flours and starches with varied functional and rheological properties. For instance, precooked flours with reduced viscosity but higher solubility can be used in preparing calorie dense gruels for complementary feeding (Harper & Jensen, 1985). The complexes that form between amylose and polar lipids improve the functional properties such as lowering the stickiness of biscuits and modifying viscosity profiles. The amylose component has been reported to increase during extrusion at low screw speeds (Sarawong, Schoenlechner, Sekiguhi, Berghofer, & Ng, 2014) because such low speeds prolongs residence time, and this increases shear to push the material through the extruder thereby increasing the amylopectin fragmentation into amylose (Moussa, Qin, Chen, Campanella, & Hamaker, 2011). This fragmentation has been postulated to impart a desirable smooth texture and may be responsible for the stable suspensions in thin porridge (Moussa et al., 2011).

Findings on effect of extrusion on oligosaccharides in cereals and legumes seem to be inconclusive. Extrusion does not seem to be effective in destroying the flatulence‐causing α‐galactosides found in cereals (Cheftel, 1986) yet starch and some oligosaccharides especially verbascose, stachyose, and raffinose was reduced in extruded kidney beans flours (Alonso et al., 2001) perhaps due to formation of starch–lipid complex (Anuonye, Badifu, Inyang, & Akpapunam, 2007). Another study reported that extrusion of beans was shown to reduce the amount of raffinose but increased the amount of stachyose (Ai, Cichy, Harte, Kelly, & Ng, 2016). More investigations are required to determine the exact effect of extrusion on cereal and legume oligosaccharides.

### 2.4 Effect of extrusion on structure of dietary fiber

Extrusion contributes to changes of physico‐chemical properties of insoluble dietary fibers leading to increase in soluble dietary fibers resulting from shearing forces (Cheftel, 1986). This is significant because the bioavailability of Fe, Cu, Zn, and Mn increases when the insoluble dietary fibers and minerals complexes are broken down to release the minerals (Cheftel, 1986). High shear–temperature combination of extrusion leads to the redistribution of insoluble dietary fiber to soluble forms (Gualberto et al., 1997). Extrusion increased the soluble fiber from 9 g/100 g to 9–14 g/100 g (Zhang, Bai, & Zhang, 2011). The largest increase was reported when extrusion was conducted at a 10% feed moisture and a temperature of 140°C indicating that extrusion at low moisture–high temperature combination favors conversion of insoluble fibers to soluble ones. A similar trend was observed in extruded wheat bran and soy (Chen, Ye, Yin, & Zhang, 2014; Yan, Ye, & Chen, 2015). In a separate study, extrusion reduced insoluble dietary fiber in oat bran and the greatest interconversion/loss of insoluble fiber was when the screw speed was low (Gualberto et al., 1997) since low screw speed brings greater compression and friction that can facilitate fiber transformation (Gualberto et al., 1997). However, at higher residence time, complexation of starch with lipids and proteins present within the food matrix can occur, leading to formation of resistant starches (Awolu et al., 2015; Sarawong et al., 2014; Vasanthan, Gaosong, Yeung, & Li, 2002) and consequently increasing soluble dietary fiber (Chanvrier et al., 2007; Huth, Dongowski, Gebhardt, & Flamme, 2000). The increase in resistant starch during extrusion can be explained in two ways. Firstly, during extrusion transglucosidation occurs where 1,4 carbon‐oxygen bonds are cleaved resulting in formation of new unhydroglucose linkages which are resistant to digestive enzymes (Stojceska et al., 2009). Secondly, there is formation of retrograded amylose which is more resistant to starch hydrolyzing enzymes (Stojceska et al., 2009; Vasanthan et al., 2002) compared to unretrograded amylose. This increase in resistant starch is of great importance in weight management as they can lower glycemic response, help in fecal elimination of bile and fats thus preventing bile recirculation (Gunnes & Gidley, 2010). In addition, their presence in the gut can illicit the production of satiety hormones and hence reduce the intake of food (Ye, Arumugam, Haugabrooks, Williamson, & Hendrich, 2015).

### 2.5 Effect of extrusion on lipids

Extrusion leads to a decrease in the extractable lipids in extruded products (Bjorck & Asp, 1983; Cheftel, 1986; Singh et al., 2007). In studies involving wheat and maize, it was reported that only 40%–55% of extractable fat in the raw materials could be extracted using ethyl ether after extrusion. This reduction could be attributed to complexing of monoglycerides and free fatty acids with amylose and protein, making them hard to extract using organic solvents (Bjorck & Asp, 1983; Singh et al., 2007). It is also possible that thermal degradation during extrusion could lead to the lower fat content of extruded products (Bjorck & Asp, 1983). This modification of lipids brings about other challenges; rancidity and oxidation are some of the concerns with extrudates containing lipids. High levels of free fatty acids resulting from hydrolysis of triglycerides can produce off‐flavors in food, reducing their shelf life. Nonetheless, extrusion denatures the hydrolytic enzymes, thereby reducing or preventing the release of free fatty acids (Camire et al., 1990). Screw wear can produce metals which are pro‐oxidants which may speed up oxidation reaction of fatty acids (Singh et al., 2007) because extrusion produces a larger surface area resulting from increased air cells found in highly expanded extrudates which increases the surface area for oxidation. For example, it was reported that extruded rice and *dhal* had higher iron content (Singh et al., 2007). Extrusion also leads to Maillard reactions which produces compounds which have antioxidants properties and therefore may reduce oxidation of lipids in extrudates (Ti et al., 2015). Additionally, lipids bound within starch are less prone to oxidation (Cheftel, 1986). Evidence does not seem to be conclusive on the effects of extrusion on lipids, implying that more studies need to be done on this area.

### 2.6 Effect of extrusion on content and bioactivity of phytochemicals

Epidemiological studies have consistently shown that consumption of food rich in phytochemicals is associated with decreased risk of developing diseases such as cardiovascular diseases, type II diabetes, obesity, and cancer (Okarter & Liu, 2010). Phytochemicals are potent antioxidants which scavenge free radicals that may cause oxidative stress. They are found embedded in food matrices thereby reducing their bioavailability (Nkhata et al., 2018). Extrusion helps disrupt the food matrix and make phytochemicals more bioaccessible (Ti et al., 2015) through increased gelatinization of starch, denaturation of protein that bind these phytochemicals, and breaking down of many complexes in which these compounds are embedded or entrapped (Wolfe & Liu, 2003). Since extrusion increases extractable amount of phytochemicals which may also be used as a proxy for increased bioaccessibility, it is not surprising that extruded rice displayed high oxygen radical absorbance capacity (ORAC) (Ti et al., 2015) because of not only increased availability of radical scavenging phytochemicals but also due to compounds formed during Maillard browning reactions that have antioxidant effect (Moreno, Fernandez, Rodriguez, Carrillo, & Rochin, 2018). The decrease in bound phenolics and increase in free phenolics observed after extrusion of rice (Ti et al., 2015) supports this notion.

The increase in extractable phenolic acid seems to be cultivar dependent. Extrusion of a blend of common beans and corn starch was reported to increase the total phenolic and antioxidant activity of extruded snacks (Anton et al., 2009). Though there was an increase in total phenolic content (TPC) and antioxidant activity in blends of corn starch and navy/small red beans, they observed that snacks prepared from red bean cultivars had higher total phenols compared to the navy beans. Upon extrusion, these workers reported that there was a 70% reduction in total phenols in red bean‐corn starch compared to the 10% loss in navy bean‐corn starch. This variation among legumes varieties is further corroborated by other researchers (Korus, Gumul, & Czechowska, 2007) who studied the trends in polyphenols such as caffeic acid, cyaniding, kaempferol, myricetin, quercetin, chlorogenic acid, ferulic acid, and p‐coumaric acid in raw and extruded beans. In red beans, they observed an increase of 14% in the extrudates compared to raw beans. This increase was accounted for by an 84% increase in quercetin and a 40% increase in ferulic acid accompanied by 33 and 9% decrease in chlorogenic and caffeic acids, respectively (Korus et al., 2007). On the other hand, black brown and cream beans recorded a 19% and 21% decrease. This affirms the proposition that cultivar type determines the effect of extrusion on bioactive compounds (Brennan et al., 2011).

Despite the increase in accessible phenolic acids, extrusion generally decrease the content of phenolic acid due to high temperature and pressure during extrusion cooking that may destroy phenolic acid (Zhang et al., 2011) which may also explain decrease in total antioxidant activity reported in polished rice (Zhang et al., 2011) and barley (Sharma et al., 2012). When the feed moisture and extrusion temperatures were increased, there was a further 8%–29% decrease in TPC and a corresponding 13%–27% decrease in total flavanoid content (TFC) (Sharma et al., 2012). In another study on whole and decorticated sorghum grains, extrusion had a significant reducing effect on total phenols and tannins for both whole and decorticated grains (Dlamini et al., 2007). A similar conclusion was reached by Gujral, Sharma, Kumar, and Singh (2012) who found out that extrusion of brown rice at 100°C led to a decrease in TPC in the range of 53%–85%. Increasing extrusion temperature to 120°C caused a further 6%–14% reduction in TPC. Decrease in TPC is attributable to destruction of heat‐labile phenolic compounds such as ferulic acid (Gujral et al., 2012), alteration in their molecular structure leading to reduction in chemical reactivity of the phenolic compounds or a certain degree of polymerization which decrease the extractability of the TPC (Sharma et al., 2012). In all these studies, the increase in antioxidant activity is positively associated with increase in free phenolic acids suggesting that free phenolic but not bound phenolic acid explains most of the increase in antioxidant properties. To ascertain the effect of extrusion on antioxidant activity, using different methods could be necessary since it has been suggested that some reagents can react with phenolic groups present in food sample (Altan, McCarthy, & Maskan, 2009) which may potentially result in overestimation of antioxidant capacity.

In summary, the antioxidant effect of phytochemicals such as polyphenols and carotenoids is associated with decreased risk of diseases that are associated with oxidative stress such as cancer and cardiovascular diseases (Belobrajdic & Bird, 2013). While these diseases are under‐diagnosed in most developing countries due to poor health systems, their low incidences in developing countries may, in part, point to the protective effect of phytochemicals since diets are predominantly whole grains which normally provide significant levels of phytochemicals (Belobrajdic & Bird, 2013). However, because of the common occurrence of undernutrition in most developing countries, programs and interventions mainly focus on alleviating undernutrition attributed to either inadequate dietary intake or reduced bioavailability of nutrients resulting from high content of antinutritional factors available in many plant‐based foods. This therefore suggests that the health promoting antioxidant properties of phytochemicals is secondary to providing nutrients in developing countries. Moreover, phytochemicals may lower the nutritional quality of the diet through mechanisms that include inhibition of digestive enzymes, binding, and complexation with minerals making them unavailable for absorption (Nkhata et al., 2018). For example, ferulic acid, a major phenolic acid in maize, inhibits α‐amylase enzymes responsible for starch digestion. Binding of polyphenols to pepsin, trypsin, and lipases is known to inhibit protein and lipid digestion (Velickovic & Stanic‐Vucinic, 2018) suggesting that presence of polyphenols in the diet may contribute to protein energy malnutrition common in developing countries. Since phytochemicals are inherent in plant food, efforts to maximize nutrient bioaccessibility by minimizing their antinutritional properties are therefore very important. The net benefit of phytochemicals to human health is, therefore, dependent on the total antioxidant effect as well as their net effect on ultimate bioavailability of nutrients and minerals. Nevertheless, the later take precedence in developing countries due to high prevalence of undernutrition.

## 3 NIXTAMALIZATION

### 3.1 Effect of nixtamalization on proteins

**Figure 4:**
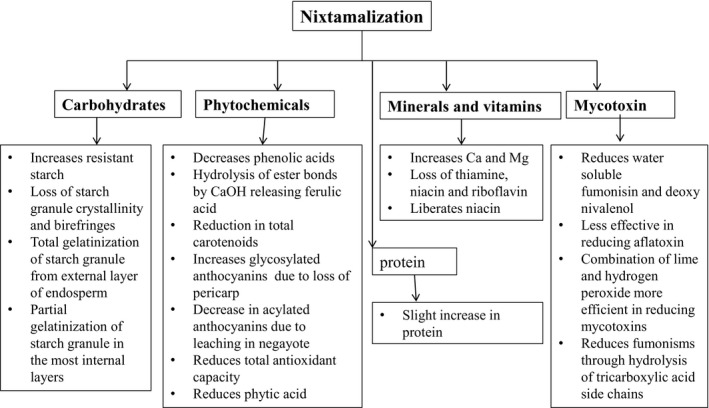
Flow diagram summarizing effects of nixtamalization on different components of cereals and grains

Figure 4 summarizes the effects of nixtamalization on substrates. Nixtamalization does not seem to have a significant impact on the protein content of grains. In a study to determine the effect of cooking maize at cooking time of 0 and 30 min, and lime concentrations of 0, 0.3, 0.5, and 1.0%, it was concluded that the process slightly enhanced the protein content from 8% to 9% due to the concentration effect (Sefa‐Dede et al., 2004), while the other study attributed the effect to the swelling of protein resulting in loss of shape (Gomez, Rooney, Waniska, & Pflugfelder, 1987).

Lime treatment of maize improves balance of essential amino acids (FAO, 1992). Imbalance of essential amino acids has been arguably suggested to be responsible for pellagragenic factor in addition to other suggestion that niacin is found in bound form in maize and therefore not available for the body (FAO, 1992). Both boiling and enzymatic digestion of maize were reported to liberate niacin from raw maize (Devika & Sherry, 2016; FAO, 1992). However, it was concluded that the difference in amino acid balance rather than in bound niacin was responsible for the difference between raw and lime processed maize in biological activity and pellagragenic action (FAO, 1992).

### 3.2 Effect nixtamalization on Carbohydrates

Nixtamalization seems to affect the shape of carbohydrates especially starch. Baking nixtamalized grains led to losses in crystallinity and birefringence of starch (Gomez et al., 1991). X‐ray diffraction and differential scanning calorimetry (DSC) showed that nixtamalization led to total gelatinization of starch granules of the external layers of the endosperm and partial gelatinization in the most internal layers (Rojas‐Molina et al., 2007). This observation is supported by the fact that the steeping water gets into contact with the external parts first and hence has longer action time on the external compared to the internal parts. The loss in crystallinity, on the other hand, is a function of the disruption of amylose and amylopectin (Rojas‐Molina et al., 2007). Gels from nixtamalized starch were softer than those from raw starch, due to partial gelatinization accrued during nixtamalization. Moreover, nixtamalization increases the content of resistant starch (Mariscal‐Moreno et al., 2017; Rendon‐Villaboles, Bello‐Pérez, Osorio‐Díaz, Tovar, & Paredes‐López, 2002) which may result in low glycemic food. However, Mendez‐Montealvo et al. (2008) reported that nixtamalization had no effect on the flow characteristics of starch dispersion.

### 3.3 Effect of nixtamalization on content and bioactivity of phytochemicals

Greater than 80% phenolic acids are found in bound form (Adom, Sorrels, & Liu, 2003). Nixtamalization resulted in hydrolysis of bound phenolics with subsequent decrease in total phenolic content in maize due to leaching out in the nejayote (Mora‐Rochin et al., 2010). Mora‐Rochin et al. (2010) reported a decrease in total phenolics after nixtamalization of maize. Similarly, De la Parra, Saldivar, and Liu (2007) reported a decrease between 35% and 44% in TPC of five maize genotypes processed through nixtamalization. These losses are enhanced by the thermal and alkaline conditions during nixtamalization, the physical loss of the pericarp, and leaching out of phenolics into the cooking liquor (Del Pozo‐Insfran, Brenes, Serna‐Saldívar, & Talcott, 2006). Ramirez‐Jimenez (2019) did not find changes in free phenolics between raw and nixtamalized corn possibly because most of the free phenolics are found in germ and germ is never removed in the subsequent steps of washing during nixtamalization unlike the pericarp (Das & Singh, 2015). Calcium hydroxide hydrolyses hemicellulose and lignin components located in germ, endosperm, and aleurone layer which are rich in phenolics (Lozovaya et al., 2000) resulting in increase in phenolic compounds in “*masa.*”

There was also reduction in flavonoids which was attributed to lixiviation into the steeping water which is usually discarded (Ramirez‐Jimenez, 2019), while ferulic and gallic acids were predominant in nixtamalized flours. It was proposed that calcium hydroxide liberates *trans*‐ferulic acid from bound ester glycosides associated with cell walls (De la Parra et al., 2007) with bound ferulic acid content reducing by 56%–83%. Ferulic acid is a potent antioxidant and is known to prevent lipid and protein oxidation (Kanaski, Aksenova, Stoyanova, & Butterfield, 2002). It constitutes up to 90% of total phenols in wheat and 98% of it is found in aleurone layer and the pericarp (Manach, Scalbert, Morand, Rémésy, & Jiménez, 2004). It is found mainly in the *trans* form esterified to arabinoxylans and hemicelluloses, and therefore, more than 90% are bound in insoluble form. In traditional nixtamalization, the lime in the steeping water can cleave the ester bond thereby releasing more ferulic acids (Carrera et al., 2012). This phenomenon explains the increase in ferulic acid during nixtamalization.

Total carotenoids diminish after nixtamalization for tortilla production (Corrales‐Banuellos et al., 2016; Mora‐Rochin et al., 2016). The reduction is attributable to effect of lime cooking coupled with thermal treatment which could have led to oxidation and isomerization of the carotenoids. Relative percentage increase was recorded in glycosylated anthocyanins (Cy‐3‐Glu and Pf‐3‐Glu) while showing an inverse effect on acylated anthocyanins (Cy‐diSuc‐Glu and Cy‐Suc‐Glu). Physical loss of the pericarp and leaching into the nejayote are some of the possible reasons for this effect (Mora‐Rochin et al., 2016, 2010).

Nixtamalization reduces antioxidant activity of grains and its derived products. The decrease emanates from reduction in total phytochemicals present in nixtamalized grains (De La Parra et al., 2007). While this effect may not seem to be a great deal because nixtamalization increases calcium and reduces mycotoxins present in kernel (Serna‐Saldivar, Almeida‐Dominguez, Gomez, Bockholt, & Rooney, 1991), nixtamalized grains might not have similar antioxidant potential as their non‐nixtamalized grains (Mora‐Rochin et al., 2010). This may seem counterintuitive as lime cooking liberates ferulic acid, the most potent antioxidant in grains, from bound ester glucosides associated with cell walls. Unfortunately, most of the liberated ferulic acid is lost in *nejayote*. Mora‐Rochín et al. (2010) compared the effect of conventional nixtamalization with extrusion cooking in terms of phytochemical retention. While both methods significantly reduced phenolic contents and antioxidant activity, nixtamalization resulted in the highest decrease due to loss of ~0.5%–14.5% into cooking liquor. The main contributory components in lost weight are pericarp, outer endosperm, and aleurone layer which are rich sources of phenolic acids (Rosentrater, Richard, Bern, & Flores, 2005). During extrusion cooking, most of these parts are retained, and therefore, both phytochemical content and antioxidant activity remain higher.

Losses of anthocyanin and *β*‐carotene were observed during nixtamalization (de Arriola, Porres, Cabrera, Zepeda, & Rolz, 1988) with a significance loss of anthocyanin of 46%‐88% due to chemical modifications at alkaline pH conditions. Anthocyanins are located in the aleurone layer and pericarp, which facilitates the removal along with steeping solution. Loss of anthocyanins was 37%, 54%, and 75% in nixtamal, tortilla, and chips, but when acidification treatment was applied, losses were reduced to 9%, 11%, and 17%, respectively (Del‐Pozo Infrans et al., 2006). A decrease by 38%–65% was reported on *β*‐carotene because of elevated temperature and chemical modifications by calcium hydroxide (De la Parra et al., 2007). Nixtamalization has also been reported to reduce lutein, zeaxanthin, *β‐*cryptoxanthin, and *β‐*carotene in yellow, red, blue, and high carotenoid maize (de la Parra et al., 2007). A significant loss of provitamin A carotenoids including *β*‐cryptoxanthin (84%) and *β*‐carotene (38%) was reported when nixtamalization was applied to produce tortilla. The loss of carotenoids in tortilla is probably due to release of carotenoids into the nixtamalization wastewater (“*nejayote*”). Rosales, Agama‐Acevedo, Bello‐Perez, Gutierrez‐Dorado, and Palacios‐Rojas (2016) investigated two methods of nixtamalization on retention of carotenoids in provitamin A biofortified maize. Compared to the control kernels that were not nixtamalized, traditional nixtamalization increased *β*‐cryptoxanthin, 9‐*cis‐ β*‐carotene, and 13‐*cis‐ β*‐carotene in the dough and tortilla probably due to loss of dry matter. There was a significant decrease in *β*‐cryptoxanthin and 9‐*cis‐β*‐carotene in nixtamalization by extrusion. In traditional nixtamalization, maize kernels were intact, while in nixtamalization by extrusion maize were processed into grits measuring 1–2 mm, which makes these compounds prone to thermal degradation, photo‐degradation, and auto‐oxidation (Rosales et al., 2016). Generally, it appears that lime cooking leads to more loss of carotenoids than subsequent processes of making tortillas or masa. This theory was supported by a study (De la Parra et al., 2007) where effects of nixtamalization on phytochemicals and antioxidant profiles of five corn varieties were reduced.

### 3.4 Effect of nixtamalization on minerals, vitamins, and antinutritional factors

Both calcium and magnesium contents increased after nixtamalization (Bressani, Turcios, & Ruiz, 2002; De la Parra et al., 2007; Devika & Sherry, 2016). Calcium increased by 94% during nixtamalization of maize (de Arriola et al., 1988). A possible reason for the increase is that calcium comes from calcium hydroxide contained in lime that is used during nixtamalization (Bressani et al., 2002; Devika & Sherry, 2016; Maria, Patricia, & Ricardo, 2010). An increase of calcium content of up to 400% had been reported in nixtamalized grains compared to non‐nixtamalized grains (Chen et al., 2014). On the other hand, the rise of magnesium is believed to come from equipment used during heating such as the cooking pans (Devika & Sherry, 2016).

It appears that wood ash used in steeping solution can promotes the intensification of potassium, zinc, and iron than lime in nixtamalized grains because the large portion of these elements present in ash solution compared to lime (Maria et al., 2010). In the same study, magnesium was reported to constantly increase with either lime or wood ash. Regarding vitamins, losses of thiamin by 60%–65% were reported in white and yellow maize likely due to degradation of kernel germ, while niacin and riboflavin were reported to decrease by 30% and 32%–52%, respectively (Devika & Sherry, 2016). Although nixtamalization reduces some of the nutritive value, it is also known to remove or reduce antinutrient factors and render nutrients free for absorption. For instance, phytic acid decreased between 4% and 28% after nixtamalization of maize (Bressani et al., 2002; Devika & Sherry, 2016). This loss of phytic acid is greater in the endosperm than in germ and is associated with increase in temperature used (Bressani et al., 2002).

### 3.5 Effect of nixtamalization on mycotoxins

Nixtamalization reduces mycotoxins that are known to be carcinogenic. During lime cooking, some mycotoxins are lost in nejayote (Abbas, Mirocha, Rosiles, & Carvajal, 1988; Schaarsmidt & Fauhl‐Hassek, 2019; Ulloa & Schroeder, 1969) especially fumonisin and deoxynivalenol which are water soluble (IARC Science, 2012). However, this may not affect aflatoxins or zearalenone which are less water soluble. Another factor that influences mycotoxin changes during nixtamalization is pH and temperature. High pH and temperatures to which grains and subsequent tortilla are subjected during lime cooking destroy some mycotoxins (Schaarsmidt & Fauhl‐Hassek, 2019) thereby making nixtamalized products low in mycotoxins. However, the effect of high pH may be reversed under acidic conditions (Mendez‐Alborez et al., 2004) especially in the stomach making it likely that aflatoxins potentially present in tortillas could become potent upon ingestion (Schaarsmidt & Fauhl‐Hassek, 2019).

Aflatoxin is a fungal metabolite, which is carcinogenic in both animals and humans can be reduced by nixtamalization. This was shown when maize inoculated with *Aspergillus flavas* and *Aspergillus parasiticus* and then subjected to lime cooking resulted in reduction in aflatoxin by 80%–100% (FAO, 1992). However, the effectiveness of lime on reducing aflatoxin is dependent on lime concentration. In a study conducted on white maize of Nutrica variety, the low level (<2.0% w/v) did not reduce aflatoxin sufficiently to make it safe for human consumption (de Arriola et al., 1988). When higher levels (2%–10%) calcium hydroxide was used, aflatoxin level had reduced significantly but still not to the allowable threshold value of 20 µg/kg. Moreover, the dough had intense yellow color with unacceptable organoleptic characteristics (de Arriola et al., 1988). This study suggests that nixtamalization has the potential of reducing aflatoxins but should be combined with other measures to reduce the levels to acceptable limits. Though these authors did not observe greater reduction in aflatoxin upon nixtamalization, the use of lime and hydrogen peroxide was reported to reduce the levels of aflatoxin B_1_ (AFB_1),_ aflatoxin M_1_ (AFM_1_), and aflatoxin B_1_‐8,9‐dihydrodiol (AFB_1_‐dihydrodiol) by 94%, 90%, and 93%, respectively (Elias‐Orozco, Castellanos‐Nava, Gaytán‐Martínez, Figueroa‐Cárdenas, & Loarca‐Piña, 2002). Mendez‐Albores et al. (2004) came to a similar conclusion when they found out that nixtamalization accounted for 92% degradation of aflatoxin B_1,_ and 90% degradation of aflatoxin B_2_ (Mendez‐Alborez et al., 2004). In summary, reduction in mycotoxins was more efficient when a combination of lime and hydrogen peroxide than when either was used. Though these findings suggest that nixtamalization is an effective way to reduce carcinogenic aflatoxins, a combination of nixtamalization and extrusion was more effective than extrusion alone (Elias‐Orozco et al., 2002).

Finally, another mycotoxin that has shown promising results in terms of its ability to be reduced during nixtamalization is Fumonisin. Fumonisin B_1_ (FB_1_) is a fungal contaminant found in maize which has been evaluated for possibility of being carcinogenic to humans (Palencia et al., 2002). Through nixtamalization, FB_1_ can be hydrolyzed, leading to loss of its tricarboxylic acid side chains. It was observed that water washed away 11% of the FB_1_ in uncooked maize. Elevation of sphigonganine, a fumonism toxicity‐related biomarker, was reduced in cells of nixtamalized tortillas (Palencia et al., 2002). Dombrink‐Kurtzman et al. (2002) affirmed this by observing that nixtamalization led to retention of only 18% of the initial concentration of fumonisin and that 75% of the fumonism was washed away. This figure is significantly higher than the 50% reported by another study (Palencia et al., 2002), and it could imply that more studies need to be done to establish the effect of nixtamalization on mycotoxins.

## 4 CONCLUSION

During extrusion and nixtamalization, many chemical changes occur that affect the chemical and nutritional composition of processed foods as summarized on Table 1. These changes include denaturation of protein, gelatinization of starch, formation of volatile flavors, increase in soluble fibers, and inactivation enzymes (Moreno et al., 2018). While the high temperature short time method used in cooking can preserve nutrients, denature antinutritional factors, and therefore increase protein digestibility compared to conventional cooking (Moreno et al., 2018), it may also decrease some bioactive compounds making extruded products low in *β*‐carotene, tocopherols, and phenolic acids and may promote Maillard browning that leads to reduction in lysine, an essential amino acid thereby reducing both the quality and quantity of protein (Hurrell, 1990). Moreover, accumulation of glycation end products (AGEs), formed from Maillard reactions, is common in extruded foods. AGEs are associated with the etiology of various diseases such as diabetes, retinopathy, and other neurodegenerative diseases (Ott et al., 2014). Although more studies need to be done to ascertain the effect of these processing methods on some nutrients such as proteins and fats, extrusion, and nixtamalization are effective processing methods for enhancement nutritional quality of cereals and legumes. While extrusion is an expensive technology for many resource poor households in many developing countries, nixtamalization is a less expensive procedure that may have similar effects on quality and safety of cereal and legume products.

**Table 1:** Summary of the effects of extrusion and nixtamalization on nutrient and phytochemical contents of cereals and legumes food products

Processing technique	Cereal/Legume	Outcomes	References
Nixtamalization			
Cooking at 95°C with 1.2% lime for 75 min, and soaking for 10 hr	Maize	Increased calcium and magnesium Decrease phytic acid Decrease in thiamin by 60%–65%, niacin by 30% and riboflavin by 32%–52%	Bressani et al. (2002) Devika and Sherry (2016) Chen et al. (2014)
Cooking with lime for 30–40 min, steeping for 12 hr	Maize	TPC decrease by 35%–45% Reduction in flavonoids Bound ferulic acid reduced by 56%–83% with consequent increase in free ferulic acid Increased zinc bioavailability Decreased antioxidant activity by 0.5%–14% Decreased anthocyanin content by 46%–88% Decreased *β*‐carotene, lutein, zeaxanthin between 38%–65% Decreased *β‐*cryptoxanthin by 84%	De la Parra et al. (2007) Carrera et al. (2012) Mora‐Rochin et al. (2010) de Arriola et al. (1988) De la Parra et al. (2007)
	Maize	Decreased aflatoxin by a range of 80%–94% Reduced levels of aflatoxin B_1_ (AFB_1),_ aflatoxin M_1_ (AFM_1_) and aflatoxin B_1_−8,9‐dihydrodiol (AFB_1_‐Dihydrodiol) by 94%, 90% and 93%, respectively Fumonism was reduced by a range of 50%–75%	FAO (1992) Schaarschmidt & Faul‐Hassek (2019) Ulloa‐Sosa and Schroeder (1969) Abbas et al. (1988) Elias‐Orozco et al. (2002) Mendez‐Alborez et al. (2004) Palencia et al. (2002)
Cooking for 30 min, lime concentration 0.33, 0.5 and 1.0%	Maize	Protein increased from 8 ‐ to 9% Insignificant increase in protein	Sefa‐Dede et al. (2004)
Extrusion			
	Peas Cereal brans	Decreased trypsin inhibitors, oxalates and phytic acid by 72%), 37%, 54%, respectively	Hejdeysz et al. (2016), Gualberto et al. (1997) Nikmaram et al. (2017)
	Wheat bran	Increased soluble fiber from 9 to 9.5–14.2 g/100g Decreased trypsin inhibitors activity (TIA)	Alonso et al. (2001) Zhang et al. (2011) Yan et al. (2015)
Temperature 115–165°C	Oat bran	Decreased insoluble fiber	Gualberto et al. (1997)
	Beans	Increased total polyphenols by 14% (84% increase for quercetin, 40% increase for ferulic acid, 33% reduction for chlorogenic acid and 9% reduction for caffeic acid) Decreased starch content Decreased stachyose, verbascose and raffinose	Korus et al. (2007) Alonso et al. (2001)
	Broken rice with wheat bran	Increased amylose Increased Fe, Cu, Ca	Sarawong et al. (2014) Singh et al. (2000)
120–150rpm, 14%–22% moisture, 140–180°C	Lentil splits	Decreased tannin by 98%, phytic acid by 99% and trypsin inhibitors 99%	Rathod and Amapure (2016)
	Chickpeas	Increased maltose, glucose and stachyose	Berrios et al. (2010)
	Soy, wheat bran	Increased glucose, galactose and stachyose Increased soluble fiber s	Yan et al. (2015) Chen et al. (2014)
Temperature at 143°C	Wheat flour	Trypsin inhibitor decreased by 57% Eliminated hemagglutinins	Bjorck and Asp (1983) Alonso et al. (2001)
	Polished rice and barley	TPC reduced by 8%–29% while total flavonoid content reduced by 13%–27%	Zhang et al. (2011) Sharma et al. (2012)
	Brown rice	TPC reduced by 53%–85%	Gujral et al. (2012)

## CONFLICT OF INTEREST

The authors declare that they do not have any conflict of interest.

## ETHICAL APPROVAL

This study does not involve any human or animal testing.
